# A systematic review of observational and report‐based measures of caregiver sensitivity: A Latin American perspective

**DOI:** 10.1002/imhj.70120

**Published:** 2026-10-24

**Authors:** Olenka Retiz, Katherine Fourment, Lucía Marquez, Magaly Noblega

**Affiliations:** ^1^ Department of Psychology Pontificia Universidad Católica del Perú Lima Peru

**Keywords:** caregiver–child interaction, Latin America, measurement, observational coding, sensitivity, systematic review

## Abstract

Sensitivity is a cornerstone of early childhood mental health; however, its measurement relies heavily on tools developed in English‐speaking, high‐resource contexts. This systematic review identified observational and report‐based instruments used to assess sensitivity in caregivers of children aged 0–6 years. It evaluated the methodological robustness and usability of these instruments within Latin American contexts. Following a systematic search in Web of Science, Scopus, PsycInfo, and Redalyc, 732 eligible studies were identified. Based on a frequency‐of‐use threshold, 16 instruments (14 observational and 2 report‐based) represented across 616 papers were selected for in‐depth evaluation. Measures were coded for theoretical alignment with Ainsworth's sensitivity definition, psychometric evidence, and usability—specifically training accessibility, administration complexity, and evidence of use with diverse Latin American populations (e.g., caregivers in rural settings or with lower educational levels). Results revealed that only a minority of measures adhered explicitly to Ainsworth's original definition. Most instruments prioritized warmth and positive affect, potentially leading to cross‐cultural underdetection in contexts where responsiveness is expressed through proximity and routine‐based care. While observational tools predominated, report‐based measures showed limited validation and minimal regional use. These findings highlight the urgent need for culturally grounded validation and the development of accessible training programs for the region.

## 1 INTRODUCTION

According to Attachment Theory, attachment refers to the affective bond that develops between an infant and their primary caregiver(s) during the first years of life and becomes organized into relatively stable patterns of security or insecurity through repeated interactions (Ainsworth, 1967; Bowlby, 1969). Secure attachment reflects the child's expectation of caregiver availability and responsiveness in contexts of stress, supporting the use of the caregiver as a secure base for exploration (Ainsworth et al., 1978).

**Key Findings**

1. Sensitivity measures often treat warmth and positive affect as central, which can miss responsive caregiving expressed through proximity and routine‐based care in some Latin American and other non‐Western contexts.
2. Many widely used observational tools require training and materials that are not available in Spanish, limiting feasibility and standardization in Latin American low‐resource settings.
3. Report‐based sensitivity measures are brief and potentially scalable but currently lack evidence linking them to attachment outcomes and observational convergence.

**Statement of relevance to infant and early childhood mental health**

Accurate assessment of caregiver sensitivity is essential for identifying relational risk and tailoring early caregiving interventions central to infant and early childhood mental health. This review clarifies how sensitivity is operationalized across common instruments and highlights when measures may under‐detect responsiveness in contexts where caregiving is expressed through proximity and daily routines. By integrating psychometric indicators with usability and Spanish‐language training access, the study supports practitioners and researchers in selecting measures that better fit real‐world service and research settings.

A substantial body of research has documented the long‐term implications of attachment security for socioemotional development and mental health. Secure attachment has been associated with greater emotional understanding and regulation, as well as more adaptive interpersonal functioning, whereas insecure attachment is linked to long‐term elevated risks of relational difficulties, externalizing behaviors, and mental health problems (Dagan et al., 2018; Deneault et al., 2023; Obeldobel et al., 2023).

In this context, caregiver sensitivity has consistently been identified as a robust predictor of secure attachment (Madigan et al., 2024). Sensitivity is defined as the caregiver's capacity to perceive, interpret, and respond appropriately to the child's signals (Ainsworth et al., 1974). Through ongoing reciprocal exchanges, sensitive caregiving supports trust in caregiver availability and the development of secure internal working models, providing a relational foundation for socioemotional competence and mental health across development (Thompson, 2016).

### 1.1 Assessing sensitivity

Given its foundational role in early socioemotional development, attachment research has increasingly emphasized the need for reliable and context‐sensitive measures of caregiving processes, particularly sensitivity (Halty & Berástegui, 2021). Observational measures are often treated as the reference approach for assessing sensitivity (Lotzin et al., 2015), as they allow direct evaluation of caregiver‐child interaction and capture sensitive behaviors that are often intuitive and automated rather than consciously planned (Mesman, 2010), making them less accessible through self‐report.

Ainsworth's Maternal Sensitivity Scale (AMSS; Ainsworth et al., 1974) is the gold standard observational measure in the study of parental sensitivity, in that it operationalizes Ainsworth's original conceptualization of sensitivity as the caregiver's prompt, appropriate, and child‐attuned responding to signals (Bretherton, 2013). Building on this framework, numerous observational instruments have been developed, differing in their conceptualization and operationalization of sensitivity (Mesman & Emmen, 2013). While some measures adhere closely to Ainsworth's original definition by focusing on contingent and contextually appropriate responses to the child's signals, others incorporate additional behavioral indicators such as warmth, verbal engagement, or structured interactional tasks (Mesman & Emmen, 2013). As Mesman (2021) highlighted, these operational choices reflect implicit assumptions about how sensitivity is expressed and have important implications for the cultural flexibility and comparability of observational measures.

Previous systematic reviews have synthesized observational measurements of parental sensitivity. Mesman and Emmen identified eight instruments most frequently used in the literature, all developed in English and primarily validated in North American or European contexts (Mesman & Emmen, 2013). A more recent review by Halty and Berástegui (2021) reported similar limitations in the linguistic and cultural scope of parental responsiveness measures, conceptualized as the behavioral manifestation of sensitivity. That review also underscored the need for more systematic evaluation of sensitivity instruments using objective, comparable criteria. To date, however, the field lacks an integrative assessment that jointly considers psychometric indicators and practical usability, including training accessibility and feasibility across diverse research settings.

Importantly, methodological rigor and practical usability do not necessarily converge in the assessment of sensitivity. Instruments with theoretical and empirical support often require extensive training, lengthy administration procedures, and specialized resources (Fourment et al., 2022), limiting their feasibility in large‐scale studies and low‐resource settings. These constraints are particularly salient in contexts such as Latin America, where access to standardized attachment measures is often restricted by financial limitations, lack of institutional infrastructure, and the high cost of training for key observational instruments, which are frequently available only in English (Mesman et al., 2016a). Conversely, instruments designed for greater feasibility may rely on narrower or behavior‐specific operationalizations of sensitivity, which can limit comparability across studies and complicate the integration of findings within a coherent theoretical framework (Mesman & Emmen, 2013).

### 1.2 Methodological challenges in Latin America

The assessment of sensitivity in Latin American research contexts presents specific methodological challenges related to the use and implementation of established measurement instruments. Cross‐national attachment research has emphasized the importance of empirically examining whether observational tools and procedures developed in Western, high‐income contexts can be validly and feasibly applied in different settings (Causadias & Posada, 2013). In Latin America, the use of sensitivity measures is often shaped by limited access to training, language barriers, and constrained research infrastructure, resulting in heterogeneous applications, partial adaptations, or reliance on a narrow set of available instruments (Fourment et al., 2022). These conditions complicate the systematic comparison of findings across studies and highlight the need to evaluate sensitivity measures not only in terms of their psychometric properties, but also in terms of their practical usability across diverse research contexts.

Beyond feasibility, there are measurement‐related reasons to question the uncritical transfer of sensitivity measures developed in Western, high‐income contexts to Latin American populations. Caregiving in Latin America often involves extended family networks, shared caregiving responsibilities, higher physical proximity, and culturally specific forms of emotional expression (Causadias & Posada, 2013; Mesman et al., 2016a). These caregiving features may not be fully captured by instruments grounded in dyadic, verbally oriented models of caregiver‐child interaction (Mesman et al., 2016b). As a result, measures that foreground overt positive affect may underestimate sensitive caregiving in non‐Western rural contexts where responsiveness is often expressed nonverbally and embedded in proximity and everyday routines (Fourment et al., 2025), thereby reducing cross‐study comparability.

Against this background, empirical research on sensitivity in Latin America remains limited. Few studies have employed established observational measures with Latin American samples (see Duhalde et al., 2020; Lima Ribeiro et al., 2021; Olhaberry et al., 2015), and those that have done so often rely on smaller samples than studies conducted in Western contexts (Fourment et al., 2022). The reliance on observational methods can be difficult to sustain in large‐scale research in the region, given the practical and institutional barriers to implementing standardized attachment methodologies in Latin America (Causadias & Posada, 2013).

One potential strategy to address these challenges may be to use report‐based measures of sensitivity. These instruments may be more feasible for large‐scale studies and for settings where observational approaches are impractical due to the substantial demands in training, time, and resources associated with observational methods (Lotzin et al., 2015). However, the systematic exclusion of report‐based measures from prior reviews has left their theoretical grounding, psychometric quality, and applicability, particularly in non‐Western contexts, largely unexplored.

### 1.3 The present study

In response to these gaps, the present study had two objectives. First, it aimed to systematically review observational and report‐based instruments used to assess sensitivity in caregivers of children aged 0–6 years, including instruments developed in English and Spanish. Second, it evaluated these instruments in terms of methodological robustness (theoretical alignment and psychometric evidence) and usability from a Latin American perspective.

Usability criteria included training accessibility, administration complexity, and evidence of use with Latin American population characteristics, including caregivers with lower educational levels and those residing in rural settings. By adopting this perspective, the study extends previous reviews focused exclusively on observational methods, English‐language instruments, and North American or European contexts (Halty & Berástegui, 2021; Mesman & Emmen, 2013).

To our knowledge, this is the first systematic evaluation of sensitivity measures integrating methodological indicators with usability and contextual relevance. Findings are intended to support researchers working in non‐Western and specifically Latin American contexts in selecting and prioritizing instruments for validation and use, advancing more inclusive and contextually grounded research on sensitivity.

## 2 METHODS

### 2.1 Search strategy

A systematic search was conducted across four scientific databases: Web of Science (WoS), Scopus, PsycInfo, and Redalyc. The search strategy followed the PRISMA guidelines for systematic reviews and meta‐analyses and aimed to identify empirical studies that used observational or report‐based measures to assess caregiver sensitivity. Prior to conducting the searches, the authors discussed and agreed upon the search terms that best captured the objectives of the review.

The search keywords used in WoS, Scopus, and PsycInfo were: (“maternal sensitiv*” OR “maternal responsive*” OR “paternal sensitiv*” OR “paternal responsive*” OR “mother* sensitiv*” OR “mother* responsive*” OR “father* sensitiv*” OR “father* responsive*” OR “parent* sensitiv*” OR “parent* responsive*” OR “sensitive parenting”) AND (child* OR infan* OR toddler OR preschooler OR baby OR babies).

Search terms were adapted for Redalyc to capture Spanish‐language publications. Spanish terms were combined using equivalent Boolean operators and parentheses to mirror the English strategy. The Spanish search terms were defined as: (sensibilidad materna* OR sensitividad* materna* OR responsividad* materna* OR sensibilidad* paterna* OR sensitividad* paterna* OR sensibilidad* parental* OR sensitividad* parental* OR responsividad* parental*) AND (infan* OR niñ* OR bebe* OR preescolar*).

Across all databases, terms were searched within the “Topic” field or its closest equivalent (title/abstract/keywords), depending on the database. Redalyc was included to capture peer‐reviewed research published in Spanish and to mitigate known language and regional coverage biases in major international databases (Mongeon & Paul‐Hus, 2016).

The publication time frame was restricted to studies published between December 8, 2012, and April 8, 2023. This time frame was selected to capture advances in the measurement of sensitivity following the systematic review conducted by Mesman and Emmen (2013). Records from disciplines not directly related to the focus of the study (e.g., broad behavioral and social sciences) were excluded.

An updated search using the same databases and search terms was conducted on May 6, 2025. For this update, the publication period ranged from April 8, 2023, to May 6, 2025.

### 2.2 Selection criteria

Studies were required to meet all of the following inclusion criteria: (a) empirical article; (b) published in English or Spanish; (c) reported the use of observational or report‐based instruments to measure caregiver sensitivity; (d) for observational studies, instruments employed macro‐coding approaches; and (e) focused on caregivers of children aged 0 to 6 years.

The age range of 0 to 6 years was selected because caregiver sensitivity plays a particularly central role in early attachment formation and emotion regulation processes during early childhood, and because many sensitivity instruments are explicitly designed and validated for this developmental period (Lotzin et al., 2015).

Studies using broader measures of parenting behavior were considered only when they included a sensitivity subscale that could be independently interpreted and evaluated (e.g., Infant/Toddler Home Observation for Measurement of the Environment [IT‐HOME]), thereby ensuring conceptual alignment with the focus of the review.

Studies were excluded if they relied on ad hoc measures, employed micro‐analytic coding systems, or if the full text was not accessible. Micro‐analytic coding approaches were excluded because their highly granular focus and context‐specific indicators limit comparability across studies and instruments, which was central to the objectives of this review. Duplicate records were removed manually.

### 2.3 Study selection process

#### 2.3.1 First search wave (2012–2023)

During the identification phase, the initial searches yielded a total of 7890 records published between December 8, 2012, and April 8, 2023, across all databases. After removing duplicate titles, 5316 records remained. During the screening phase, records were excluded if they were published in languages other than English or Spanish, did not assess sensitivity, focused on children older than 6 years, or did not report empirical results. This reduced the number of records to 767.

During the eligibility phase, full‐text articles were reviewed, and 150 records were excluded due to lack of full‐text access, use of ad hoc measures, or reliance on micro‐analytic coding. This resulted in a final sample of 617 articles meeting the inclusion criteria.

#### 2.3.2 Second search wave (2023–2025)

In the identification phase of the updated search, 3923 records were retrieved. After removing duplicates, 2396 records remained. Screening exclusions followed the same criteria as in the first search wave, resulting in 199 records. During the eligibility phase, full‐text review led to the exclusion of studies without accessible full text, those using ad hoc measures, or those employing micro‐analytic coding. This resulted in a final sample of 115 articles.

The study selection process is summarized in Figure 1, following the PRISMA flow diagram (Moher et al., 2009). The diagram integrates the identification, screening, eligibility, and inclusion steps across both search waves, treating records retrieved in the initial and updated searches as a single combined corpus.

**FIGURE 1:**
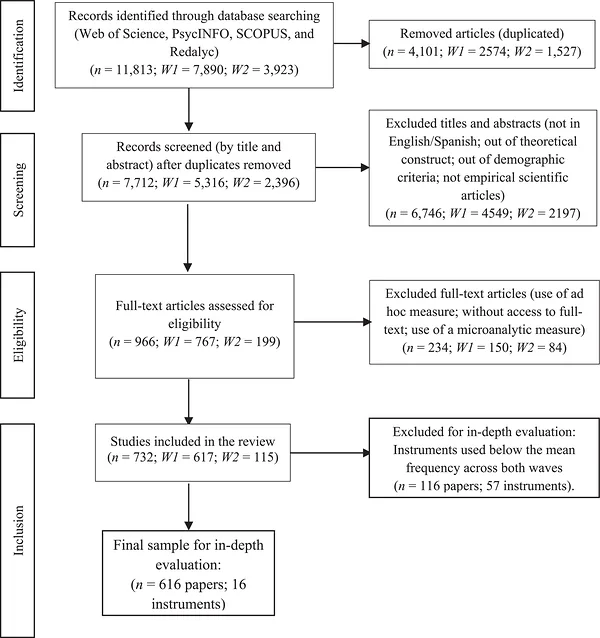
Prisma flow diagram. *Note. W1* = First search wave (2012–2023); *W2* = Second search wave (2023–2025). The diagram integrates the identification, screening, and eligibility steps across both search waves, resulting in a combined corpus of 732 eligible studies. From this total,16 instruments (14 observational and 2 report‐based) were selected for in‐depth evaluation based on a frequency‐of‐use threshold. These 16 instruments were represented in 616 of the included papers.

To ensure the reliability of the study selection process, during the first search wave, the first author screened and coded all records according to the predefined inclusion and exclusion criteria, and the third author independently screened and coded a random 20% subsample. Percentage agreement exceeded 95%, reflecting a high level of concordance. Disagreements were resolved through discussion within the research team. During the second search wave, screening and coding were conducted exclusively by the first author.

It bears clarifying that this review follows a measurement‐focused systematic review design, in which the unit of analysis is the instrument rather than the individual study. Accordingly, quality assessment was conducted at the instrument level—drawing on information extracted across all studies using each instrument—rather than through a study‐level appraisal tool. This design choice reflects the primary aim of the review, which was to evaluate the psychometric and usability profile of instruments, not to synthesize effect sizes or draw causal inferences from individual studies. This approach is consistent with similar instrument‐focused systematic reviews in the psychometric literature (e.g., Halty & Berástegui, 2021; Mesman & Emmen, 2013).

### 2.4 Selection of instruments for in‐depth evaluation

To identify the subset of instruments for in‐depth evaluation, we focused on the most frequently utilized measures across the combined corpus of 732 papers (resulting from both search waves). We operationalized “use” at the study‐sample level: when a paper reported multiple independent samples, each was counted as a separate use; otherwise, each paper contributed a single use. This distinction was made to avoid underestimating the prevalence of instruments frequently employed in multi‐study reports. We acknowledge that alternative thresholds, such as percentile cutoffs, could yield slightly different selections.

Within the observational and report‐based categories separately, we first identified all unique instruments used in the corpus. We then computed the arithmetic mean of uses per instrument within each category. Instruments were retained for in‐depth coding only if their frequency of use exceeded the category‐specific mean. This pragmatic threshold was selected to prioritize instruments with sustained uptake and established presence in the literature while maintaining a feasible sample for exhaustive data extraction.

It should be noted that this frequency‐based threshold introduces a potential selection bias: instruments with lower uptake—including locally developed measures, tools published in non‐indexed regional venues, or more recently developed report‐based instruments—may have been excluded despite possessing relevant psychometric or usability properties. This limitation is particularly salient for report‐based measures, which, as noted in the Introduction, may offer practical advantages for research in Latin American and other low‐resource contexts. Findings should therefore be interpreted with the recognition that the instruments evaluated here represent the most visible segment of the sensitivity measurement literature, not necessarily its full scope.

### 2.5 Data extraction

Data extraction was conducted independently by the first and third authors. For each instrument, all studies employing the instrument were reviewed to extract information on the following characteristics: number of items, response options, administration time, scoring time, requirement for official training (yes/no), training cost and duration, availability of validation studies, countries in which the instrument has been used, and characteristics of caregiver samples. It is worth noting that validation studies were defined a priori as publications whose primary aim was psychometric evaluation (e.g., reliability, validity, or internal structure), rather than substantive studies that only applied the instrument or reported ancillary measurement indices.

For the purposes of this review, Latin American samples were defined as studies conducted in Latin American countries (Central/South America and Mexico). Studies conducted in the United States or Europe with Latin American‐origin participants were not classified as Latin American samples.

Information on reliability (internal consistency, test–retest reliability, and intraclass correlation) and validity (content, construct, and internal structure validity) was extracted when available. When such information was not reported in published articles but was indicated to be accessible upon request, the authors contacted the instrument developers directly.

Throughout the Results section, information derived directly from the reviewed corpus of studies is distinguished from information obtained through other sources. Where data were obtained from instrument manuals, developers' official websites, or direct author contact rather than from reviewed studies, this is indicated explicitly in the text (*e.g., “based on information available on the instrument's official website” or “confirmed through direct author contact”*).

### 2.6 Quality and usability assessment

Evaluation criteria differed for observational and report‐based instruments. Criteria are presented in Table 1.

**TABLE 1:** Instrument evaluation criteria.

Criterion	Observational measures	Report based measures
Quality assessment		
Sensitivity definition	Sensitivity is defined according to Ainsworth et al. (1974)	
Validation studies	Has at least one validation study published.	
Reliability	At least 75% of studies report adequate inter‐rater reliability (≥.70).	At least 75% of studies report adequate internal consistency reliability (≥.70).
Criterion validity (Child attachment outcomes)	At least 75% of those studies that have hypothesized a relationship with measures of attachment have found it.	
Convergent validity	–	Has evidence of convergent validity with observational sensitivity measures (≥.30).
Content validity	–	Has evidence of content validity.
Construct validity	–	Reports an internal structure model with good fit indices obtained by an exploratory factor analysis (mean communality ≥. 60, factor number and composition coherent with theoretical proposal) or confirmatory factor analysis (TLI and CFI ≥ .95; SRMR < .08, RMSEA ≤ .06).

Usability assessment		
Rural samples	Has been used with rural populations	
Low‐educated samples	Has been used with poorly educated samples.	
Latin American samples	Has been used with Latin American samples.	
Accessible training	When formal certification is required, training is offered remotely and is available in Spanish. For instruments without formal certification, manuals or essential training materials are officially provided in Spanish.	–
Short administration time	Administration takes 15 min or less.	Has 25 items or less.
No need for a laboratory	Does not require using a laboratory.	–

*Note*: Criteria were scored as present (1) or absent (0). A total of 10 criteria were applicable to observational instruments and 11 to self‐report instruments, as indicated by non‐applicable cells (–). Different reliability standards were applied to reflect the nature of each measurement approach: inter‐rater reliability was prioritized for observational instruments to ensure consistency between independent coders, whereas internal consistency was used for report‐based measures to evaluate the internal coherence of self‐reported items.

Each criterion was scored as present (1) or absent (0), and scores were summed to provide a descriptive index of how many criteria were met. It should be noted that binary scoring does not capture gradations in evidence strength—such as the number of studies meeting a criterion, the diversity of contexts in which an instrument has been used, or the proportion of Latin American studies within a given instrument's corpus. The scores are intended as a structured descriptive tool to facilitate comparison, not as definitive rankings.

For the accessible training criterion, training was considered formally available in Spanish when at least one of the following conditions was met: (a) the instrument developers' official website explicitly documented Spanish‐language training or certification pathways; (b) published manuals or training materials in Spanish were identified through the systematic search or through direct contact with instrument developers; or (c) developers confirmed the availability of Spanish‐language training in response to author inquiries. Remote availability of training was noted but was not considered sufficient to meet the criterion in the absence of Spanish‐language materials. We acknowledge that informal or locally organized training pathways may exist, but could not be systematically verified through the sources available to this review.

These totals are intended to support structured comparison across instruments under the present operational definitions. They are not intended as definitive rankings, do not assume equal importance across criteria, and should not be interpreted as global indicators of “best” instruments in the abstract. For observational instruments, total scores ranged from 0 to 10. For report‐based instruments, total scores ranged from 0 to 11.

## 3 RESULTS

### 3.1 Search and selection results

The systematic search and selection process narrowed an initial pool of 73 identified instruments (60 observational and 13 report‐based) down to a final sample of 16 measures: 14 observational and 2 report‐based. Although these 16 instruments represent a fraction of the total identified tools, they accounted for the vast majority of the literature, being collectively used in 616 papers (approximately 84% of the total included studies).

The general characteristics and measurement approaches of these instruments are summarized in Table 2.

**TABLE 2:** Instruments included in the systematic review.

Instrument	Child's age (in months)	Measurement approach	Specific sensitivity domain?	Sensitivity domain(s) measured	Number of ratings or items (total/sensitivity‐related)	Instrument original language	Number of papers
Observational measures							
AMSS	3–24	Global rating scale	No	–	9/9	English	110
CARE‐Index	2–70	Observational coding system	Yes	Sensitivity	7/1	English	47
CIB	2–36	Observational coding system	Yes	Parental sensitivity	33/5	English	24
EAS	0–60	Observational coding system	Yes	Sensitivity	6/1	English	112
Erickson Scales	12–36	Global rating scale	Yes	Support presence	7/2	English	29
E.S.A.	6–36	Observational coding system	Yes	Empathic response, playful interaction, emotional expression	19/19	Spanish	13
GRS	2–12	Global rating scale	Yes	Sensitivity	44/1	English	28
IT‐HOME	0–36	Observational coding system	Yes	Responsivity	45/11	English	15
MBQS	0–36	Q‐sort observational	No	–	90/90	English	48
MBPQS	36–71	Q‐sort observational	No	–	90/90	English	13
Mini‐MBQS	0–36	Q‐sort observational	No	–	25/25	English	32
NICHD Scales	6–36	Observational coding system	Yes	Sensitivity	8/3	English	142
PCIRS	6–36	Observational coding system	Yes	Sensitivity	16/1	English	14
PICCOLO	10–47	Observational coding system	Yes	Responsiveness	29/7	English	13
Report‐based measures							
BCHAPS	0–5	Report‐based questionnaire (rated by nurse)	No	–	13/13	English	3
MIRI	0–12	Self‐report questionnaire	No	–	18/18	English	10

*Notes*: “Child's age” refers to the age range of children included in the studies in which the instrument was used, rather than the age range for which the instrument was originally designed. “Specific sensitivity domain” indicates whether the instrument includes a distinct sensitivity subscale or explicitly operationalized sensitivity dimension.

Abbreviations: AMSS, Ainsworth's Maternal Sensitivity Scale (Ainsworth et al., 1974); BCHAPS, Boston City Hospital Assessment of Parental Sensitivity (Zahr & Cole, 1991); CARE‐Index, Child‐Adult Relationship Experimental Index (Crittenden 1989, CIB, Coding Interactive Behavior (Feldman, 1998); EAS, Emotional Availability Scales (Biringen et al., 1998); Erickson Scales, Erickson Scales (Erickson et al., 1985); E.S.A., Escala de Sensibilidad del Adulto (Santelices et al., 2012); GRS, Global Rating Scales of Mother‐Infant Interaction (Murray et al., 1996); IT HOME, Infant Toddler Home Observation (Caldwell & Bradley, 2001); MBQS, Maternal Behavior Q‐Sort (Pederson & Moran, 1995); MBPQS, Maternal Behavior for Preschoolers Q‐sort (Posada et al., 2007); Mini MBQS, Mini‐Maternal Behavior Q‐Sort (Moran, 2009); MIRI, Maternal Infant Responsiveness Instrument (Amankwaa et al., 2007); NICHD Scales, National Institute of Child Health and Human Development Sensitivity Scales; PCIRS, Parent‐Child Interaction Rating Scales (Sosinsky et al., 2004); PICCOLO, Parenting Interactions with Children: Checklist of Observations Linked to Outcomes (Roggman et al., 2013).

### 3.2 Descriptive characteristics of the instruments

In this section, descriptive characteristics of the included instruments are reported according to their measurement approach.

#### 3.2.1 Observational measures

Of the 14 observational instruments included, three assess caregiver sensitivity using holistic or global approaches. Specifically, Ainsworth's Maternal Sensitivity Scale (AMSS), Erickson Scales, and Global Rating Scales of Mother‐Infant Interaction (GRS) rely on global rating scales, in which the evaluator observes caregiver–child interactions and assigns global judgments of sensitivity or related dimensions. In contrast, three others, Maternal Behavior Q‐sort (MBQS), Maternal Behavior for Preschoolers Q‐sort (MBPQS), and Mini‐Maternal Behavior Q‐Sort (Mini‐MBQS), employ an observational Q‐sort approach, whereby the caregiver's behavioral profile is compared to a sensitivity prototype.

The remaining eight observational instruments are based on observational coding systems, which require the assessment of multiple behaviors or interactional dimensions that are subsequently aggregated to yield a total sensitivity score or sensitivity subscore: Child‐Adult Relationship Experimental Index (CARE‐Index), Coding Interactive Behavior (CIB), Emotional Availability Scales (EAS), Escala de Sensibilidad del Adulto (E.S.A.), Infant Toddler Home Observation (IT‐HOME), National Institute of Child Health and Human Development Sensitivity Scales (NICHD Scales), Parent‐Child Interaction Rating Scales (PCIRS), and Parenting Interactions with Children: Checklist of Observations Linked to Outcomes (PICCOLO).

In terms of instrument application, most observational instruments have been used primarily to assess maternal sensitivity, although nearly all have also been applied to fathers, except for IT‐HOME. Additionally, EAS, NICHD Scales, and E.S.A. have been used with non‐parental caregivers, such as educational staff or other caregiving agents.

With respect to child characteristics, all observational instruments except E.S.A. have been used in non‐typical development samples, such as neurodevelopmental conditions and other clinical conditions.

Notably, E.S.A. is the only observational instrument included in the review that was originally developed in Spanish.

#### 3.2.2 Report‐based measures

The report‐based measures included are unidimensional instruments designed to assess caregiver sensitivity without structured observational procedures. The Maternal Infant Responsiveness Instrument (MIRI) is a self‐report questionnaire completed by caregivers, whereas the Boston City Hospital Assessment of Parental Sensitivity (BCHAPS) is rated by health professionals based on their clinical appraisal of parental behavior.

Both instruments conceptualize sensitivity as a global construct without subscales. Furthermore, MIRI and BCHAPS have been used to assess sensitivity in mothers and fathers, primarily in clinical contexts, and have been applied in samples of caregivers of preterm infants, children at developmental risk, or infants hospitalized in neonatal intensive care units.

### 3.3 Quality assessment criteria

In this section, results corresponding to the Quality Assessment criteria are presented, following the previously specified domains.

#### 3.3.1 Sensitivity definition

Only three of the 14 observational instruments explicitly define sensitivity in accordance with the original definition proposed by Ainsworth et al. (1974): AMSS, NICHD Scales, and PICCOLO. The remaining instruments incorporate core components of sensitivity, such as caregiver availability and contingent responsiveness to child signals, but extend the construct to include, to varying degrees, indicators of warmth and positive affect during interaction. In some instruments, such as EAS and CARE‐Index, these elements play a substantial role in sensitivity assessment, whereas in others, such as the PCIRS, they are less emphasized. Across instruments, warmth and positive affect are typically operationalized through behaviors such as smiling, animated tone of voice, physical affection, or verbal expressions of appreciation.

Among report‐based measures, MIRI conceptualizes sensitivity in close alignment with Ainsworth's definition, emphasizing the caregiver's ability to interpret infant signals and respond appropriately. In contrast, BCHAPS includes items assessing positive affect in interaction as well as the caregiver's capacity to modify behavior according to health professionals’ recommendations, incorporating elements that are not fully aligned with the original definition of sensitivity.

#### 3.3.2 Validation studies

Nine of the 14 observational instruments have at least one published validation study. No formally published psychometric validation studies were identified for the PCIRS (only unpublished thesis data were found), NICHD Scales, GRS, Erickson Scales, and CARE‐Index. Both report‐based instruments included in the review have published validation studies.

#### 3.3.3 Reliability

Regarding reliability, 12 out of the 14 observational instruments met the predefined criterion, with at least 75% of studies reporting adequate inter‐rater reliability (≥.70). IT‐HOME did not meet this criterion because inter‐rater reliability was frequently not reported. While E.S.A. studies did report inter‐rater reliability, less than 75% of these reports achieved values ≥.70, thus failing to meet the predefined adequacy threshold.

When reported, inter‐rater reliability was most commonly estimated using the Kappa coefficient or the intraclass correlation coefficient (ICC), depending on the instrument and design.

For report‐based instruments, studies using MIRI reported a mean internal consistency coefficient of .85, whereas studies using the BCHAPS reported a mean internal consistency of .95. In all cases, internal consistency was estimated using Cronbach's alpha.

#### 3.3.4 Criterion validity (child attachment outcomes)

For eleven of the observational instruments for which studies hypothesized a relationship between sensitivity and child attachment, at least 75% of those studies confirmed the hypothesized association. No studies using PCIRS or PICCOLO hypothesized or tested the relationship between sensitivity and child attachment. In the case of the Erickson Scales, evidence did not reach the predefined 75% criterion.

None of the report‐based instruments were used in studies that hypothesized a relationship between sensitivity and child attachment.

#### 3.3.5 Content validity

Both report‐based instruments demonstrated evidence of content validity, as documented through item development processes that included expert judgment by professionals with expertise in caregiving, sensitivity, and measurement.

#### 3.3.6 Convergent validity

None of the report‐based instruments included in this review reported evidence of convergent validity with observational measures of sensitivity.

#### 3.3.7 Construct validity

No published evidence of construct validity based on exploratory or confirmatory factor analyses was identified for the report‐based instruments.

### 3.4 Usability assessment criteria

In this section, results corresponding to the Usability Assessment criteria are presented, following the domains previously specified.

#### 3.4.1 Rural samples

Eleven out of the fourteen observational instruments have been used with rural samples, except for the Erickson Scales, MBPQS, and E.S.A. Among the instruments used in rural contexts, MBQS and AMSS have been applied in Andean and Amazonian Peruvian samples (e.g., Fourment et al., 2021, 2025), and MBQS has been used in rural Colombia (Barone et al., 2021). Notably, this criterion was coded based on sample descriptors reported across the full international corpus of studies, not exclusively in Latin American contexts. None of the report‐based instruments have been used in rural samples.

#### 3.4.2 Low‐educated samples

All instruments, both observational and report‐based, have been used in studies including caregivers with low levels of formal education.

#### 3.4.3 Latin American samples

Based on country‐of‐application information extracted from studies conducted in Latin American countries, eleven observational instruments have been used with Latin American samples. The observational instruments not identified in Latin American studies were the Erickson Scales, NICHD Scales, and PCIRS. To illustrate the regional evidence base by instrument: E.S.A. was originally developed in Chile and has been applied across multiple Chilean studies (e.g., Santelices et al., 2015; Woldarsky et al., 2019); CARE‐Index has been used extensively in Chile (e.g., Binda et al., 2019; Farkas et al., 2017; Muzard et al., 2021; Olhaberry et al., 2022)and has also been applied in Brazil (da Costa Ribeiro et al.,^2020^),; AMSS has been applied in Peruvian rural samples and in Brazilian and Chilean samples (Coo et al.,^2024^; Fourment et al., 2021, 2025; Lima Ribeiro et al., 2021); MBQS has been used in Peruvian, Colombian, and Chilean samples (e.g., Brahm et al., 2016; De Jesús Ossa Henao et al., 2023; Nóblega et al., 2016); PICCOLO has been applied in Brazilian and Uruguayan contexts (Alves et al., 2022; Balsa et al., 2024); and MBPQS has been used in Peruvian and Ecuadorian studies (Díaz Mosquera & Nóblega, 2020; Nóblega et al., 2019, 2023).

None of the report‐based instruments have been used with Latin American samples. It is important to note that, for all other usability criteria (rural samples, low‐educated samples, administration time, and laboratory requirement), evidence was drawn from the full international corpus and does not directly reflect feasibility within Latin American contexts specifically.

#### 3.4.4 Accessible training

All observational instruments require some form of training to ensure appropriate use or to achieve acceptable inter‐rater reliability, as documented in instrument manuals and developers' official training materials. Among instruments requiring formal certification, training is predominantly offered in English, based on information available on developers' official websites and training platforms at the time of data extraction. Although certification for several widely used measures (e.g., EAS, CIB, and AMSS) is available in remote formats, these instruments did not meet the criterion because Spanish‐language training/certification is not formally documented as available through these sources.

In contrast, among certification‐based instruments, CARE‐Index, MBQS, and MBPQS met this criterion because Spanish‐language training/certification pathways are formally documented on developers' official websites or were confirmed through direct author contact with instrument developers. The Mini‐MBQS did not meet this criterion, as no formal Spanish‐language training or materials were identified through these sources.

For instruments that do not require formal certification and rely on self‐paced training, such as the Erickson Scales and GRS, manuals and core training materials were identified as available only in English through official sources and therefore do not meet the accessibility criterion. Similarly, the NICHD Scales, PCIRS, IT‐HOME, and PICCOLO did not meet the criterion, as no formally available Spanish‐language manuals or training materials were identified through the sources consulted, including official websites, published documentation, and author inquiries.

#### 3.4.5 Short administration time

Administration of eleven of the fourteen observational instruments involved video recordings of caregiver‐child interactions lasting around 10 min. In most studies, coding time was not reported. Administration tends to be more time‐intensive for Q‐sort instruments (MBQS and MBPQS) and for IT‐HOME.

Both report‐based instruments include fewer than 25 items, suggesting relatively brief administration.

#### 3.4.6 No laboratory requirement

All observational instruments can be administered outside laboratory settings, and report‐based instruments do not require laboratory infrastructure.

### 3.5 Summary of combined scores

Among observational instruments, AMSS obtained the highest number of fulfilled quality criteria, whereas PCIRS met the fewest quality criteria according to the predefined framework. With respect to usability, the CARE‐Index met the largest number of usability criteria, while the Erickson Scales and IT‐HOME met fewer usability criteria. When quality and usability criteria were considered jointly, several instruments showed comparatively broader coverage across both domains, including AMSS, CIB, EAS, Mini‐MBQS, and PICCOLO.

Among report‐based instruments, none met all quality criteria. Relative to the BCHAPS, the MIRI fulfilled a greater number of criteria across both quality and usability domains.

A comparative summary of the evaluation scores for all instruments is provided in Tables 3 and 4.

**TABLE 3:** Results of the instrument evaluation for observational measures.

Measure	Quality assessment criteria				Usability assessment criteria						Overall score
	Sensitivity definition	Reliability	Validation studies	Criterion validity	Rural samples	Low‐ED samples	LA samples	Accessible training	Short time	No laboratory	
AMSS	1	1	1	1	1	1	1	0	1	1	9
CARE‐Index	0	1	0	1	1	1	1	1	1	1	8
CIB	0	1	1	1	1	1	1	0	1	1	8
EAS	0	1	1	1	1	1	1	0	1	1	8
Erickson Scales	1	1	0	0	0	1	0	0	1	1	5
E.S.A.	0	0	1	1	0	1	1	1	1	1	7
GRS	0	1	0	1	1	1	1	0	1	1	7
IT‐HOME	0	0	1	1	1	1	1	0	0	1	6
MBQS	0	1	1	1	1	1	1	1	0	1	8
MBPQS	0	1	1	1	0	1	1	1	0	1	7
Mini MBQS	0	1	1	1	1	1	1	0	1	1	8
NICHD Scales	1	1	0	1	1	1	0	0	1	1	7
PCIRS	0	1	0	N/A	1	1	0	0	1	1	5
PICCOLO	1	1	1	N/A	1	1	1	0	1	1	8

*Note*: Criteria were scored as present (1) or absent (0). N/A = not applicable (no studies using the instrument assessed child attachment security, therefore the criterion could not be evaluated).

**TABLE 4:** Results of the instrument evaluation for report‐based measures.

Measure	Quality assessment criteria							Usability assessment criteria				Overall score
	Sensitivity definition	Reliability	Validation studies	Criterion validity	Convergent validity	Content validity	Construct validity	Rural samples	Low‐ED samples	LA samples	Short time	
BCHAPS	0	1	1	N/A	0	1	0	0	1	0	1	5
MIRI	1	1	1	N/A	0	1	0	1	1	0	1	7

*Note*: Criteria were scored as present (1) or absent (0). N/A = not applicable (no studies using the instrument assessed child attachment security, therefore the criterion could not be evaluated).

## 4 DISCUSSION

This systematic review examined sensitivity measures through the joint lens of psychometric quality indicators and practical usability. Our findings point to recurring patterns in how sensitivity is defined, operationalized, and may be made implementable in research settings, particularly in Latin American contexts.

### 4.1 Conceptual heterogeneity and implications for construct validity

A central finding was the conceptual heterogeneity in how sensitivity is defined and operationalized. Only a small group of observational measures explicitly adhered to Ainsworth's definition of sensitivity as the caregiver's capacity to perceive, interpret, and respond contingently to the child's signals (Ainsworth et al., 1974). This pattern is consistent with prior work showing substantial heterogeneity in how sensitivity is operationalized across instruments and settings (Mesman, 2021; Mesman & Emmen, 2013).

Most instruments extended the construct by incorporating indicators of warmth and positive affect expression. Although warmth and positive affect often co‐occur with sensitive caregiving (Davidov & Grusec, 2006; Fields‐Olivieri et al., 2017; Smith & Stephens, 2018), their systematic inclusion as core indicators in the instruments may shift sensitivity toward broader indices of parenting quality, potentially increasing construct overlap and reducing comparability across studies that use different operational definitions (Mesman & Emmen, 2013). Importantly, when sensitivity is operationalized primarily through overt positive affect, there is a potential risk to underdetect the construct in contexts where contingent responsiveness is expressed more through proximity, instrumental support, and routine‐based caregiving than through explicit verbal or affective displays (Fourment et al., 2025; Mesman et al., 2016a). This does not imply that warmth is irrelevant; rather, it highlights how indicator selection can differentially weight culturally salient expressions of responsiveness, with implications for measurement equivalence in cross‐cultural comparisons (Causadias & Posada, 2013).

### 4.2 Evidence accumulation does not follow a validation‐first logic

A second pattern emerging from this review is that evidence accumulation in the sensitivity measurement field does not follow a “validation first, widespread use later” sequence. Some instruments with a large uptake across the corpus did not meet the present validation‐study criterion, whereas other measures with fewer uses did have at least one published validation study. Importantly, this pattern partly reflects our conservative operational definition of validation studies, which privileges explicit psychometric papers and may undercount dispersed validation evidence reported within substantive studies.

This discrepancy suggests that uptake is shaped not only by formal psychometric efforts but also by field‐level dynamics such as legacy status, training networks, and instrument availability. Under the present framework, this creates a practical challenge for researchers as widely used instruments may offer comparability advantages, yet the published record may still be limited with respect to explicit validation studies as operationalized here, particularly when validation evidence is embedded across heterogeneous substantive papers rather than reported in dedicated psychometric publications.

### 4.3 Methodological indicators and usability as partially independent dimensions

A third pattern was the partial independence between methodological indicators and usability. Instruments differed in whether stronger conceptual alignment and psychometric evidence co‐occurred with usability.

This matters for study design. Measures that require extensive training or time‐intensive administration may yield high‐quality observational data but constrain sample sizes and scalability. More feasible approaches may allow wider coverage but require stronger validation programs to ensure theoretical alignment and measurement precision. These trade‐offs may shape which samples are feasible to recruit, which caregiving practices are represented in the literature, and which forms of evidence accumulate over time.

### 4.4 Training, language, and structural barriers to measurement equity

Although many instruments have been used with low‐educated samples and several have been applied in Latin America, Spanish‐accessible training and materials were uncommon under the present definition. Among the instruments with documented Latin American use, only CARE‐Index, MBQS, and MBPQS met the accessible training criterion, as Spanish‐language certification pathways are formally available for these tools. This is notable given that CARE‐Index and MBQS account for a substantial portion of the Latin American corpus identified in this review, including studies conducted in Chile, Peru, and Colombia (e.g., Barone et al., 2021; Binda et al., 2019; Fourment et al., 2021; Nóblega et al., 2016; Olhaberry et al., 2022). In contrast, widely used instruments such as EAS, AMSS, and NICHD Scales—for which Latin American applications were also identified—do not currently offer formally documented Spanish‐language training, limiting the degree to which researchers in the region can implement them with full standardization. This matters because language and training infrastructures shape who can implement instruments with adequate standardization and reliability, especially in low‐resource contexts (Fourment et al., 2022; Mesman et al., 2016a). From a measurement perspective, limited access to training materials in the languages of target contexts can function as a structural barrier that contributes to uneven geographic coverage and may perpetuate the underrepresentation of Global South research in cumulative evidence bases.

Beyond training access, the usability criteria evaluated in this review point to specific features that may facilitate cultural integration in Latin American contexts. Instruments requiring brief, naturalistic observation in home or community settings—such as AMSS, Mini‐MBQS, and CARE‐Index—may be better suited to the practical realities of Latin American research than tools requiring laboratory infrastructure or lengthy certification processes. Similarly, instruments that do not constrain the caregiving context to structured play tasks may better capture the proximity‐based and routine‐embedded forms of responsiveness documented in some Latin American populations (Fourment et al., 2021, 2025). Culturally grounded adaptation studies are needed to test whether core indicators of sensitivity function equivalently across these contexts, and to identify which usability features are most relevant for researchers and practitioners in the region.

### 4.5 Observational salience and the underdevelopment of report‐based measures

Observational methods were predominant in the measurement of caregiver sensitivity in the reviewed literature. As noted before, observational approaches are often treated as the reference standard for sensitivity assessment because they capture interactional processes that may not be fully accessible to caregiver report (Lotzin et al., 2015; Mesman, 2010).

Report‐based measures were rarely used in the corpus and showed a narrower evidence base relative to observational instruments. They were also absent from rural samples and rarely used in Latin American contexts. Under the present criteria, report‐based instruments showed adequate internal consistency and evidence of content validity, but no evidence of convergent validity with observational measures, and no use in attachment‐outcome studies. These gaps do not imply that report‐based approaches are inherently inadequate. Rather, they indicate that the current literature does not support strong claims of equivalence with observational assessment or of attachment‐related validity. This is particularly relevant for large‐scale studies and low‐resource settings, where observational approaches may be difficult to implement due to training and time demands (Fourment et al., 2022; Lotzin et al., 2015). A clear next step is targeted validation programs that test convergence with observational measures and examine criterion validity with attachment‐related outcomes.

### 4.6 Implications for further research and limitations

Taken together, findings point to an apparent mismatch between widely used sensitivity measures and the implementation realities of research in Latin America. Although several observational instruments have been applied in the region and many have been used with low‐educated samples, language‐accessible training was uncommon. This creates a barrier to standardization and scalability and may constrain both the geographic scope and the diversity of populations included in sensitivity research.

From a Latin American perspective, measures that capture proximity‐based, activity‐oriented, but not necessarily warm expressions of responsiveness may provide stronger construct validity in some populations. Yet these expressions appear underweighted in many widely used instruments. Without explicit attention to these issues, cross‐cultural comparisons risk reflecting measurement artifacts rather than substantive differences in caregiving.

Future research should prioritize three directions. First, report‐based instruments require systematic validation, including convergent validity with observational measures and attachment‐related validity. Second, culturally grounded adaptation studies are needed to test whether core indicators of sensitivity function equivalently across contexts, and to identify culturally salient manifestations of contingent responsiveness. Third, greater transparency regarding training requirements, language accessibility, and resource demands is needed to reduce structural barriers and promote measurement equity.

This work has limitations. We focused on the most frequently used instruments based on a frequency‐of‐use threshold, which may have excluded locally developed measures, regionally published tools, or emerging report‐based instruments with emerging relevance. This is particularly notable for report‐based measures, which may offer practical advantages in Latin American and other resource‐constrained settings but remain underrepresented in international databases. In addition, the review relied primarily on information that was publicly available through published articles, manuals, and formally documented training materials. Although the authors contacted instrument developers directly when information on psychometric properties or training procedures was indicated as available upon request, most of these inquiries did not receive a response. Consequently, additional validation evidence, adaptations, or training pathways may exist but could not be verified or included in the present evaluation. Informal or locally developed training pathways may also exist, but could not be systematically assessed.

As this review follows a measurement‐focused systematic review design, study‐level quality appraisal was not conducted. This is a recognized limitation when PRISMA guidelines are applied to instrument‐focused reviews, and future work may benefit from integrating study‐level quality indicators to complement instrument‐level evaluation.

Furthermore, criteria were coded dichotomously to support transparent comparison, but this approach does not capture gradations in evidence strength (e.g., number of validation studies, breadth of contexts represented, or the proportion of Latin American studies within a given instrument's corpus). Except for the Latin American samples criterion, usability criteria were assessed using the full international corpus and do not directly reflect feasibility within Latin American contexts. Future work could complement this framework with weighted scores, evidence tiers, or criteria operationalized specifically within regional corpora.

## 5 CONCLUSION

By jointly evaluating methodological indicators and usability, this review highlights structural patterns in how caregiver sensitivity is measured. Under the present criteria, no single instrument optimized conceptual clarity, psychometric evidence, and usability across contexts. Advancing the field will require a more context‐sensitive and pluralistic approach to measurement, alongside infrastructures that support training and standardization beyond English‐language and high‐resource settings.

## FUNDING

This work was supported by the Pontificia Universidad Católica del Perú through the Research Support Fund (Fondo de Apoyo a la Investigación, FAI‐034‐2023).

## CONFLICT OF INTEREST STATEMENT

The authors declare no conflicts of interest.

## PARTICIPANT CONSENT STATEMENT

Participant consent was not required, as this study is a systematic review of previously published research and did not involve direct data collection from human participants.

## DIVERSITY AND ANTI‐RACIST SCHOLARSHIP STATEMENT

This review reflects an appreciation for diversity and an anti‐racist approach by critically examining how systems of knowledge production, training access, and language shape the measurement of caregiver sensitivity in infant and early childhood mental health. Rather than treating measurement tools as culturally neutral, the review explicitly considers the countries and contexts in which instruments have been developed, validated, and applied, with particular attention to Latin American contexts characterized by linguistic diversity, rurality, and structural resource constraints. Because this study synthesizes published research rather than collecting new human participant data, racial and ethnic characteristics are described through contextual indicators permitted in the source studies, including country of study, caregiver education level, and rural or urban context. The review intentionally cites and integrates work by scholars based in Latin America and other non–high‐income contexts and foregrounds structural barriers to training and standardization as equity‐relevant measurement issues. In doing so, it aims to support more inclusive, context‐sensitive assessment practices in infant and early childhood mental health research.

## Data Availability

Data supporting the findings of this study are available from the corresponding author upon reasonable request.
